# Use of technologies by nurses to promote breastfeeding: a scoping review

**DOI:** 10.1590/1980-220X-REEUSP-2022-0466en

**Published:** 2024-02-02

**Authors:** Maria Sauanna Sany de Moura, Simone Barroso de Carvalho, Zeila Ribeiro Braz, Loisláyne Barros Leal, Ana Maria Ribeiro dos Santos, Marcia Teles de Oliveira Gouveia, Fernanda Valéria Silva Dantas Avelino, Ana Roberta Vilarouca da Silva

**Affiliations:** 1 Universidade Federal do Piauí, Departamento de Pós-Graduação em Enfermagem, Teresina, PI, Brazil.; 2 Universidade Federal do Piauí, Departamento de Graduação em Enfermagem, Picos, PI, Brazil.

**Keywords:** Nurses, Male, Technology, Breast Feeding, Health Services, Enfermeros, Tecnología, Lactancia Materna, Servicios de Salud, Enfermeiros, Tecnologia, Aleitamento Materno, Serviços de Saúde

## Abstract

**Objective:**

To map evidence on technologies used by nurses to promote breastfeeding in Health Services.

**Method:**

This is a scoping review, based on the recommendations of the Joanna Briggs Institute and following the PRISMA Extension for Scoping Reviews, carried out in 2022. The searches took place in seven databases, using the following combined descriptors: “nurse”, “technology”, “breastfeeding”, and “health services”.

**Results:**

Fifteen articles were found, the first from 2000 and the last from 2022, all published in English with a predominance of productions in the United States of America (n = 5) and Brazil (n = 3). The link was the technology present in most studies (n = 11). However, with regard to classification, educational and hard technologies were the most used in promotion strategies (n = 14 and n = 12), respectively.

**Conclusion:**

The articles showed a variety of technologies used to promote breastfeeding in health services, and thus, contributing for the maintenance and duration of breastfeeding.

## RESUMO

**Objetivo:**

Mapear evidências sobre tecnologias utilizadas por enfermeiros para promoção do aleitamento materno nos Serviços de Saúde.

**Método:**

Trata-se de uma revisão de escopo, baseada nas recomendações do Joanna Briggs Institute e seguindo o PRISMA Extension for Scoping Reviews, realizada no ano de 2022. As buscas aconteceram em sete bases de dados, utilizando-se os seguintes descritores combinados: “nurse”, “technology”, “breastf feeding” e “health services”.

**Resultados:**

Foram encontrados 15 artigos, sendo o primeiro de 2000 e o último de 2022, todos publicados em língua inglesa com predominância de produções nos Estados Unidos da América (n = 5) e no Brasil (n = 3). O vínculo foi a tecnologia que esteve presente na maioria dos estudos (n = 11). Porém, no que se refere à classificação, as tecnologias educacionais e duras foram as mais utilizadas nas estratégias de promoção (n = 14 e n = 12), respectivamente.

**Conclusão:**

Os artigos mostraram uma variedade de tecnologias usadas para a promoção do aleitamento materno nos serviços de saúde, e assim, contribuindo para a manutenção e duração do aleitamento materno.

## RESUMEN

**Objetivo:**

Mapear evidencia sobre tecnologías utilizadas por enfermeros para promover la lactancia materna en los Servicios de Salud.

**Método:**

Esta es una revisión de alcance, basada en las recomendaciones del Instituto Joanna Briggs y siguiendo la Extensión PRISMA para Revisiones de Alcance, realizada en 2022. Las búsquedas se realizaron en siete bases de datos, utilizando los siguientes descriptores combinados: “nurse”, “technology”, “breastf feeding”e “health services”.

**Resultados:**

Se encontraron 15 artículos, el primero del año 2000 y el último del año 2022, todos publicados en inglés con predominio de producciones de Estados Unidos de América (n = 5) y Brasil (n = 3). El vínculo fue la tecnología que estuvo presente en la mayoría de los estudios (n = 11). Sin embargo, en cuanto a la clasificación, las tecnologías educativas y duras fueron las más utilizadas en las estrategias de promoción (n = 14 y n = 12), respectivamente.

**Conclusión:**

Los artículos mostraron una variedad de tecnologías utilizadas para promover la lactancia materna en los servicios de salud y, así, contribuir para el mantenimiento y duración de la lactancia materna.

## INTRODUCTION

The baby’s proper nutrition is essential for proper growth and development. Child malnutrition is an important public health problem, as it is associated with worse morbidity, mortality, and low health outcomes in the long term^(1)^. Thus, breastfeeding constitutes an ideal practice for infant feeding^(2)^, given that human milk, in addition to its nutritional value, has immunological and microbiological properties that are important for the baby’s health^(3)^.

Among the benefits of breastfeeding, we can mention the improvement of the baby’s development^(4)^, the reduction of the risk of infant mortality, the increased protection against infectious and chronic diseases, hydration, and strengthening of the emotional bonds in the mother-baby binomial^(5)^. In this regard, adequate maternal guidance regarding the advantages of breastfeeding constitutes an important strategy for strengthening this practice.

Therefore, given the epidemiological scenario and the benefits arising from breastfeeding for the mother-child binomial, the need to enhance actions to promote, protect, and support breastfeeding emerges^(6)^. The literature shows that among the resources for promoting this practice are health technologies, which can contribute to breastfeeding through education and strengthening of the bond and dialogue between health professionals and service users^(7)^.

In the context of health, these resources are classified into three categories, according to their density. They are soft technologies, which consist of the relationships and bonds established between workers and users of health services; soft-hard, which cover the knowledge necessary for clinical reasoning; and hard, which include machines, equipment, and medicines used in healthcare^(8)^.

Thus, with regard to health services, especially Primary Health Care, which is the gateway to the user, the relevance of developing and improving soft technologies has been emphasized, such as embracement, strengthening of autonomy, and valuing of the individuals’ subjectivity for the construction of a health education space, favoring, among others, the practice of breastfeeding^(9)^.

Also from this perspective, in the context of nursing, three categories of technologies are discussed. They are: educational, managerial, and assistance technologies, which fulfill relevant functions in the production of care. Educational technologies can and should be used in any and all educational spaces, formal and informal ones, inside and outside nursing care. In relation to management technologies, they constitute an important tool to be used in the health service, given that they allow the development of a bond based on dialogue among individuals. With regard to care technologies, these are actions developed in nursing care in a systematic way, using instruments, methods, and theories aimed at producing quality care for human beings in all their dimensions^(10)^.

Nursing care technologies can be defined as the result of the articulation of scientific knowledge for the production of material goods, or not, used during intervention in everyday practical situations and/or within the scope of research, seeking to resolve human and structural health-related issues. In this respect, they encompass any innovative strategies that can be used to facilitate the promotion, protection, and recovery of health^(10)^.

The act of breastfeeding, although perceived in many cultures as instinctive, requires specific health care targeted at the needs of the binomial. Therefore, the introduction of technologies to promote breastfeeding can contribute to reducing barriers to this practice, such as insufficient knowledge, discontinuity of care, and unsatisfactory support, factors that interfere with the initiation and maintenance of breastfeeding^(2)^.

Given the importance of breastfeeding for the mother-child binomial and the limitations found in its implementation, the rationale of this research is the need to investigate the existing resources used by nurses in breastfeeding assistance. The relevance of this study lies on the possibility of gathering evidence that can guide and support health professionals in making decisions regarding the use of each intervention in different breastfeeding support contexts.

In view of the above, the objective of this study is to map evidence on the technologies used by nurses to promote breastfeeding in Health Services.

## METHOD

### Design of Study

This is a scoping review, based on the method proposed by the Joanna Briggs Institute (JBI), which aims to provide an overview of the evidence, as well as identify and analyze knowledge gaps and inform future research. Thus, the steps proposed by JBI were followed, which included: the definition of the objective and research question; the development of inclusion criteria; the description of the approach to search, selection, data extraction, and presentation of evidence; search; selection; extraction; analysis of evidence; results presentation; and summary of evidence^(11)^.

The review question was prepared based on the Population, Concept, and Context (PCC) strategy, with the following being defined: P – nurse; C – technology/ breast feeding; C - health services. Taking that into account, the guiding question was formulated: What are the technologies used by nurses to promote breastfeeding in Health Services?

The protocol for this scoping review is registered with Open Science Framework at the link: https://osf.io/aynbt/. The research was carried out from August to October 2022, according to the JBI scoping review methodology, in the databases Medical Literature Analysis and Retrieval System (PubMed/MEDLINE), Latin American and Caribbean Literature in Health Sciences (LILACS) via Virtual Health Library (VHL), Cumulative Index to Nursing and Allied Health Literature (CINAHL) via Ebsco, Excerpta Medica Database (EMBASE), Scopus via Elsevier, and Nursing Database (BDENF) via VHL. The Scientific Electronic Library Online (SciELO) virtual library was also accessed. The search strategies described in Chart 1 were developed according to each database chosen and in accordance with the Health Sciences Descriptors (DeCS) and Medical Subject Headings (MeSH), in the month of October 2022.

**Chart 1:** Database search strategies with Boolean operators – Teresina, Piauí, Brazil, 2022.

Base	Strategy	Records recovered
PubMed	(((“Nurses”[Mesh] OR (Nurse) OR (Personnel, Nursing) OR (Nursing Personnel) OR (Registered Nurses) OR (Nurse, Registered) OR (Nurses, Registered) OR (Registered Nurse)) AND (“Technology”[Mesh] OR (Industrial Arts) OR (Arts, Industrial))) AND (“Breast Feeding”[Mesh] OR (Breastfed) OR (Breastfeeding) OR (Breast Fed) OR (Milk Sharing) OR (Sharing, Milk) OR (Breast Feeding, Exclusive) OR (Exclusive Breast Feeding) OR (Breastfeeding, Exclusive) OR (Exclusive Breastfeeding) OR (Wet Nursing))) AND (“Health Services”[Mesh] OR (Health Service) OR (Services, Health))	98
LILACS	((Nurses) OR (Nurse) OR (Personnel, Nursing) OR (Nursing Personnel) OR (Registered Nurses) OR (Nurse, Registered) OR (Nurses, Registered) OR (Registered Nurse)) AND ((Technology) OR (Industrial Arts) OR (Arts, Industrial)) AND ((Breast Feeding) OR (Breastfed) OR (Breastfeeding) OR (Breast Fed) OR (Milk Sharing) OR (Sharing, Milk) OR (Breast Feeding, Exclusive) OR (Exclusive Breast Feeding) OR (Breastfeeding, Exclusive) OR (Exclusive Breastfeeding) OR (Wet Nursing)) AND ((Health Services) OR (Health Service) OR (Services, Health))	02
CINAHL	(MH “Nurses” OR “Nurse” OR “Personnel, Nursing” OR “Nursing Personnel” OR MH “Registered Nurses” OR “Nurse, Registered” OR “Nurses, Registered” OR “Registered Nurse”) AND (MH “Technology” OR “Industrial Arts” OR “Arts, Industrial” AND MH “Breast Feeding” OR “Breastfed” OR “Breastfeeding” OR “Breast Fed” OR “Milk Sharing” OR “Sharing, Milk” OR “Breast Feeding, Exclusive” OR “Exclusive Breast Feeding” OR “Breastfeeding, Exclusive” OR “Exclusive Breastfeeding” OR “Wet Nursing”) AND (MH “Health Services”OR “Health Service” OR “Services, Health”)	23
EMBASE	(‘nurses’/exp OR ‘nurses’ OR ‘nurse’/exp OR ‘nurse’ OR ‘personnel, nursing’ OR ‘nursing personnel’/exp OR ‘nursing personnel’ OR ‘registered nurses’ OR ‘nurse, registered’ OR ‘nurses, registered’ OR ‘registered nurse’/exp OR ‘registered nurse’) AND (‘technology’/exp OR ‘industrial arts’ OR ‘arts, industrial’) AND (‘breast feeding’/exp OR ‘breastfeeding’/exp OR ‘breastfed’ OR ‘breast fed’ OR ‘milk sharing’ OR ‘sharing, milk’ OR ‘breast feeding, exclusive’ OR ‘exclusive breast feeding’ OR ‘breastfeeding, exclusive’ OR ‘exclusive breastfeeding’ OR ‘wet nursing’) AND (‘health services’ OR ‘health service’/exp OR ‘services, health’)	05
Scopus	(TITLE-ABS-KEY (“Nurses” OR “Personnel, Nursing” OR “Nursing Personnel” OR “Registered Nurses” OR “Nurse, Registered” OR “Nurses, Registered” OR “Registered Nurse”) AND TITLE-ABS-KEY (“Technology” OR “Technology” OR “IndustrialArts” OR “Arts, Industrial”) AND TITLE-ABS-KEY (“Breast Feeding” “Breast Feeding” OR “Breastfed” OR “Breastfeeding” OR “Breast Fed” OR “Milk Sharing” OR “Sharing, Milk” OR “Breast Feeding, Exclusive” OR “Exclusive Breast Feeding” OR “Breastfeeding, Exclusive” OR “Exclusive Breastfeeding” OR “Wet Nursing”) AND TITLE-ABS-KEY (“Health Services” OR “Health Service” OR “Services, Health”))	10
BDENF	((Nurses) OR (Nurse) OR (Personnel, Nursing) OR (Nursing Personnel) OR (Registered Nurses) OR (Nurse, Registered) OR (Nurses, Registered) OR (Registered Nurse)) AND ((Technology) OR (Industrial Arts) OR (Arts, Industrial)) AND ((Breast Feeding) OR (Breastfed) OR (Breastfeeding) OR (Breast Fed) OR (Milk Sharing) OR (Sharing, Milk) OR (Breast Feeding, Exclusive) OR (Exclusive Breast Feeding) OR (Breastfeeding, Exclusive) OR (Exclusive Breastfeeding) OR (Wet Nursing)) AND ((Health Services) OR (Health Service) OR (Services, Health))	01
**Virtual Health Library** SciELO	((Nurses) OR (Nurse) OR (Personnel, Nursing) OR (Nursing Personnel) OR (Registered Nurses) OR (Nurse, Registered) OR (Nurses, Registered) OR (Registered Nurse)) AND ((Technology) OR (Industrial Arts) OR (Arts, Industrial)) AND ((Breast Feeding) OR (Breastfed) OR (Breastfeeding) OR (Breast Fed) OR (Milk Sharing) OR (Sharing, Milk) OR (Breast Feeding, Exclusive) OR (Exclusive Breast Feeding) OR (Breastfeeding, Exclusive) OR (Exclusive Breastfeeding) OR (Wet Nursing)) AND ((Health Services) OR (Health Service) OR (Services, Health))	00

Source: Prepared by the authors.

### Selection Criteria

For the selection of studies, the inclusion criteria were primary, empirical, quantitative, and qualitative articles of any design or methodology that present technology to promote breastfeeding used by nurses in health services, in all languages, with no publication deadline. The exclusion criteria were: those that did not answer the research question and studies that were not available in full for free.

### Data Collection

**Figure 1:**
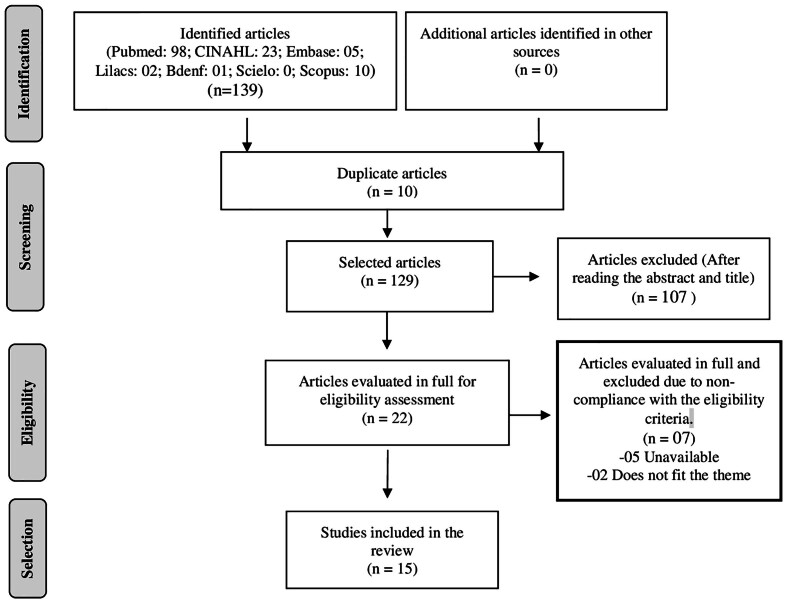
Preferred Reporting Items for Systematic Reviews and Meta-Analyses Extension for Scoping Reviews (PRISMA-SCR) flowchart on study selection. Teresina, Piauí, Brazil, 2022.

The study selection process was carried out by two independent reviewers, and disagreements were resolved by a third reviewer. The selection of studies was carried out in two stages. First, the titles and abstracts of references identified through the search strategy were evaluated, and potentially eligible studies were pre-selected. In the second stage, the full text of the pre-selected studies was evaluated to confirm their eligibility (Figure 1). The selection of studies according to title and abstract was carried out using the digital tool Rayyan QCR^(9)^. The articles selected from each database were imported in the BibTex file format. Subsequently, two reviewers independently and blindly read the titles and abstracts to reduce the possibility of interpretative bias. Then, a third reviewer proceeded with the evaluation of the articles that presented divergences to define the studies inclusion or exclusion. In cases where doubts about the selection remained, progress was made to the next stage, corresponding to the full reading.

To present the selection process of scoping review studies, the Preferred Reporting Items for Systematic Reviews and Meta-Analyses extension for Scoping Reviews (PRISMA ScR) flowchart was used, according to JBI recommendations^(1)^.

To extract data from the selected studies, an instrument made available by JBI and adapted for this study was used^(1)^. The information selected was: title; year of publication; country; language; study approach; population; strategies carried out, and technologies used.

### Data Analysis and Treatment

Data analysis was performed with mapping of information collected by the instrument used in this study. A descriptive analytical framework was used to examine the text of each article. In consideration of that, a qualitative analysis of all content was carried out, which allowed the creation of categories that emerged from the more in-depth analysis of the publications, which were able to illustrate the topics of interest, with the categorization of findings into: soft, soft-hard, hard, educational, managerial, and assistance.

When processing the data, only peer-reviewed publications were considered. A critical evaluation of the texts was also carried out, mainly regarding the method, according to the reviewers’ expertise.

## RESULTS

The search in the databases mapped 139 potentially eligible studies, with 15 remaining in the final sample, as shown in Figure 1.

Regarding the characteristics of the 15 selected studies, the first was published in 2000 and the others, discontinuously, in 2022. The largest production occurred in 2018, with three articles, followed by 2008, 2009 and 2015 with two articles in each year. In addition to the nursing professionals involved in the use of technologies to promote breastfeeding, other professional categories also contributed to the actions, such as pediatricians, obstetricians, psychologists, and nutritionists.

The countries where the studies were produced were: United States of America (n = 5), Brazil (n = 3), China (n = 2), Australia (n = 2) and, with one article each, England, Ireland and Israel. All were published in English, with eleven studies of quantitative approach, three with a qualitative approach, and one with mixed approach.

Regarding the population studied, seven studies were conducted with mothers and babies, three with pregnant women, two with healthcare professionals, and the other studies included nursing students, parents, and people with visual impairments with one article each. The technologies used for this promotion were: bond (n = 11), telephone (n = 4), video (n = 3), application (n = 2), online platform (n = 1), website (n = 1), course (n = 1), tablets (n = 1), DVD (n = 1), and humming *cordel* literature (n = 1). Some studies used more than one technology as a strategy to promote breastfeeding.

Chart 2 shows the characteristics of the publications according to the strategies for promoting breastfeeding and the technologies used for this promotion.

**Chart 2:** Characterization of selected articles according to year of publication, strategies for promoting breastfeeding, and the technologies used for this promotion – Teresin, Piauí, Brazil, 2022.

Article title	Year	Promotion strategy	Technology used/	Technology category
The baby friendly breast-feeding management course^(12)^.	2000	Course for healthcare professionals aimed at increasing knowledge, skills, and positive attitudes towards breastfeeding, as well as confidence to actively support breastfeeding mothers and their babies.	18-hour lactation course/Bonding	– Soft-hard/soft – Educational – Managerial
Evaluation of breastfeeding websites for patient education^(13)^.	2006	Breastfeeding websites for patient education, evaluated based on Health Information Technology Institute (HITI) criteria, readability, and eight content criteria from the American Academy of Pediatrics (AAP) policy statement on breastfeeding	Websites	– Hard – Educational
Prenatal breastfeeding education and breastfeeding outcomes^(14)^.	2008	Prenatal education about breastfeeding. There was a class using video demonstration and group teaching by a lactation consultant, a support group for new mothers with one-on-one teaching, prenatal and weekly postpartum meetings, led by a lactation consultant and a pediatrician. Prenatal breastfeeding education can influence the amount of time women breastfeed.	Video/Bond	– Hard/soft – Educational – Managerial
Related factors in using a free breastfeeding hotline service in Taiwan^(15)^.	2008	The use of a free hotline service for breastfeeding mothers in Taiwan. This strategy includes categories of common breastfeeding problems at different stages after birth.	Telephone/Bond	– Soft – Educational – Managerial
Breastfeeding promotion for infants in neonatal units: a systematic review and economic analysis^(16)^.	2009	Evaluate the efficacy and cost-effectiveness of interventions that promote or inhibit breastfeeding or breast milk feeding for babies admitted to neonatal units.	Bond	– Soft – Managerial
[Creation of an assistive technology for validation among blind people: focus on breastfeeding]^(17)^.	2009	Assistive health technology to encourage breastfeeding, aiming to contribute to a more autonomous life for people with disabilities and, at the same time, promote the inclusion of these people. This constructed technology is a *cordel* to be hummed by *repentistas* to later be appreciated by people with visual impairment.	Humming *cordel*/Bond	– Soft-hard/soft – Educational – Managerial
[Nursing technologies to promote breast feeding: integrative literature review]^(18)^.	2011	Video/filming was the technology most used by nurses to promote breastfeeding and most were classified as hard technology.	Video/filming	– Hard – Educational
Developing and testing an online breastfeeding training among undergraduate nursing students^(19)^.	2015	Online training for undergraduate nursing students. The development of this training included consultation with content and technology experts. The online component consisted of five modules with a combined duration of approximately 16 hours. After training, students increased their levels of knowledge related to breastfeeding.	Online platform	– Hard – Educational
Incorporating breastfeeding education into prenatal care^(20)^.	2015	Prenatal education about breastfeeding. Three breastfeeding modules were created and offered to women at prenatal consultations at 32, 34 and 36 weeks using tablets. Implemented in an obstetric clinic, women successfully learned breastfeeding content through the tablet methodology.	Tablets/Bond	– Hard/soft – Educational – Managerial
A randomized controlled trial of the effectiveness of a breastfeeding training DVD on improving breastfeeding knowledge and confidence among healthcare professionals in China^(21)^.	2018	Recruitment of healthcare professionals carried out in three hospitals in Zhejiang Province, China, in 2014 for breastfeeding DVD training to improve their knowledge and confidence in breastfeeding support skills.	DVD	– Hard – Educational
popular and inexpensively printed booklets or pamphlets containing folk novels, poems and songs^(22)^	2018	Educational interventions delivered by telephone to pregnant or breastfeeding women on the duration and exclusivity of breastfeeding.	Telephone/Bond	– Hard/soft – Educational – Managerial
Can a Call Make a Difference? Measured Change in Women’s Breastfeeding Self-efficacy Across Call Interactions on a Telephone Helpline^(23)^.	2018	Helplines that provide 24-hour contact with specialists and nurses for women facing challenges with breastfeeding. This strategy serves for interaction about breastfeeding, building self-efficacy.	Telephone/Bond	– Hard/soft – Educational – Managerial
Telelactation via Mobile App: Perspectives of Rural Mothers, Their Care Providers, and Lactation Consultants^(24)^.	2019	(CoTelelactation via cell phone app in rural Pennsylvania. Direct-to-consumer (DTC) telelactation using two-way video via personal devices has the potential to increase access to Internationally Certified Lactation Consultants (IBCLCs) in rural areas that do not have them. It is an acceptable model for rural women with limited access to in-person breastfeeding support.	Application/video/Bond	– Hard/Hard/ – Soft – Educational – Managerial
Supporting, failing to support and undermining breastfeeding self-efficacy: Analysis of helpline calls^(25)^.	2020	24-hour telephone support line for parents with Queensland nursing staff as reactive support. This reactive bonding strategy was effective in providing immediate responses at a time that may pose a risk of interruption in breastfeeding.	Telephone/Bond	– Hard/soft – Educational – Managerial
Smartphone-based counseling and support platform and the effect on postpartum lactation: a randomized controlled trial^(26)^.	2022	Daily smartphone-based feedback and a counseling platform between postpartum women and a multidisciplinary team. Counseling was provided via an app specifically developed by a multidisciplinary team (obstetricians, nurses, lactation counselors, and psychologist) in an attempt to assist and advise on maintaining lactation.	Application/Bond	– Hard/soft – Educational – Managerial

Source: PRISMA-SCR^(27)^.

The technologies that emerged from the scoping review used to promote breastfeeding were categorized based on the analysis of all strategies mentioned in the selected articles. It was possible to categorize the technologies according to theoretical classification^(10,28)^, implemented and evaluated in health services. Chart 3 contains the technology classifications^(10,28)^.

**Chart 3:** Distribution of studies according to technology classifications according to the theoretical framework adopted^(10,28)^ – Teresina, Piauí, Brazil, 2022.

Authors	Classification of technologies	Technologies used to promote breastfeeding	References of articles with the technology used according to Chart 2
Merhy^(28)^	– Soft	Bond	(12,14,15,16,17,20,22,23,24,25,26)
	Soft/hard	Course Humming cordel	(12,17)
	Hard	Site Video Telephone no. Online platform Tablets DVD Application	(13,14-18-24,15-22-23-25,19,20,21,24-26)
Nietsche et al.^(10)^	Educational	Course Humming *cordel* Site Video Telephone no. Online platform Tablets DVD Application	(12,17,13,14-18-24,15-22-23-25,19,20,21,24-26)
	Managerial	Bond	(12,14,15,16,17,20,22,23,24,25,26)
	Assistance	–	–

Source: Prepared by the authors.

Regarding the categorization of technologies, hard technology was the most used in studies (n = 12), followed by soft technology (n = 11). The analysis also revealed that educational technology is the one that has been most applied in health services (n = 14), followed by managerial technology (n = 11). No assistive technology was mentioned in the selected studies.

## DISCUSSION

Food is crucial for all stages of life and especially in the early years, as it is essential for growth, development, solidification of habits, and preservation of health. Over the years, there were efforts focused on promoting advances and implementing public policies aimed at promoting, protecting, and encouraging breastfeeding. However, challenges are still constant and must be overcome to ensure a healthy and adequate diet early in life^(5)^.

In Brazil, even with the increase in breastfeeding rates, the duration is still shorter than recommended, while two out of every three children under the age of six months receive other types of milk, especially cow’s milk, normally with the addition of some type of flour or sugar, noting that only one child continues to be exclusively breastfed until six months^(5)^.

Thus, all actions aimed at encouraging the practice of exclusive breastfeeding until six months of life are required, given their essentiality, and it is in this context that the use of technologies gains space and has been widely discussed in health services, to strengthen the importance of breastfeeding, with the use of these tools by health professionals, especially nurses, being quite common to engage the target audience^(29)^.

It is clear that care actions supported by a technological apparatus mediate the care provided more effectively, by promoting greater organization and meaningful learning, with these devices being at the same time accredited as a process and product^(18)^. This way, technology must cover the entire development of the health work process, which goes from the primordial idea, elaboration, implementation to the results^(28)^.

The relevance of promoting greater scientific knowledge regarding the topic of breastfeeding and the use of care technologies allowed discussions regarding the findings in this study which, through a deeper analysis of the contents of the publications, led to the categorization of technologies in soft, soft-hard, and hard^(28)^.

Furthermore, among the resources used by nurses to promote breastfeeding, the prevalence of technologies classified as hard, followed by the soft ones, was identified in the studies. Therefore, it is highlighted that hard technologies help to encourage desired behavior, by favoring the dissemination of knowledge and encouraging self-care practices. Moreover, the nurse must be a constant collaborator in the development, application, and evaluation of learning^(18)^, especially on the topic of breastfeeding, where mothers constitute the main target audience, considering that the main factors that lead to the interruption of breastfeeding are related to difficulties experienced by them.

However, it is worth highlighting that hard technologies are more effective when the knowledge is mediated by a health professional, aiming at encouraging discussions^(18)^ on the objective reality in which we wish to intervene. This opens up space for the development of critical and reflective thinking, as well as for the protagonism of actions on the part of the target audience.

Although hard technologies are widely used to develop strategies to encourage breastfeeding, one cannot forget that soft technologies, in the field of nursing, take on the dimension of care itself, as they use assumptions specific to human relationships, essential to establishing a bond, when considering actions such as dialogue, listening, sharing of knowledge, perception of health needs, empathy, among other aspects covered in the holistic view of care^(30)^.

Furthermore, in breastfeeding, establishing a bond in human relationships is fundamental; therefore, the support provided to women during the breastfeeding process goes beyond a group of techniques^(18)^. Thus, technology cannot only incorporate the format of a tangible product, but also be the result of abstract actions in favor of a specific objective, in this case the promotion of breastfeeding^(28)^.

The use of soft technologies must be constant, known, and promoted in health services^(31)^, due to the importance of establishing a bond between the nursing professional and the client and the perception of the inherence of interpersonal care as something substantial^(32)^. Therefore, the health sector must work to promote the insertion of soft technologies, as well as the conscious use of technologies classified as hard, with the aim of not breaking with the ideals of assistance aimed at humanized care^(33)^.

Therefore, soft technologies, as they promote humanization and the establishment of bonds between team and client, strengthen and qualify the work process, on the pillars of the development of critical and reflective thinking, encouraging the individuals’ autonomy and protagonism, in a process mediated by communication and qualified listening^(34)^.

Moreover, in this study, there was no emphasis on the soft-hard technology, as its use was verified in only two studies in the sample^(12,17)^. A review study^(18)^ on the types of technologies used by nurses to promote breastfeeding found in its results that no article in the sample used soft-hard technology. However, in a randomized and controlled clinical trial carried out with 104 postpartum women, in which this technological modality was used, it was noticed that the intervention group had a higher percentage of exclusive breastfeeding than the control group (p < 0.05), as this type of technology provides verbal, visual, and tactile stimuli, mediated by dialogue and subjectivity, which end up positively influencing learning and building practical experiences^(35)^, reaffirming the need for more studies in this area.

According to the literature analyzed, it is clear that the nursing staff has been applying, in most cases, hard technology as a resource to promote breastfeeding, and the use of other modalities should also be encouraged, especially soft-hard, where lesser use was noticed in this study, highlighting the importance of incorporation by nurses of care practice, given that scales, instruments, theories, methods and processes, such as the nursing process, provide scientific basis and ensure support for professional practice^(18)^.

It is worth highlighting that actions aimed at promoting breastfeeding must involve constant professional involvement, given the extreme importance of breastfeeding for the mother-child binomial^(5)^. Furthermore, the establishment of innovative strategies that use technological resources in the field of health education with the aim of promoting learning and strengthening preventive and timely behaviors to promote breastfeeding is of great value^(35)^.

From the analysis of the studies, the classification of technologies into educational, managerial, and assistance also emerged as a categorization^(10)^. In this research, those classified as educational prevailed. Innovating in educational actions, which encourage adherence and continuation of breastfeeding for a longer period of time, constitutes an additive to overcoming the barriers involved in women’s and children’s health, without disregarding the social support network^(35)^. Thus, the importance of developing and implementing strategies that assist nurses in transmitting the knowledge necessary to manage breastfeeding becomes increasingly evident.

The studies belonging to the sample of this scoping review did not include the use of technologies classified as assistance, which is characterized as a gap, considering the relevance of this technological modality to support the practice of breastfeeding, especially through the application of scales^(29)^.

Regarding the teaching-learning process mediated by the use of different technologies, it is important to infer that each person is unique in presenting specific individualities and particularities, and the professional must be sensitive to this. Thus, he will make a precise distinction between the most appropriate resource for each reality, so that there is greater understanding and acceptance on the part of the individual. This way, the technology will sensitize a greater number of people and with greater quality, to be verified by quantifying the expected results.

Professional training is an important management technology, which must be prioritized when applying a technological resource to a specific audience, as it will result in obtainment of the expected results from its use, being a great scenario for working on strategies aimed at continuing education.

In view of this analysis, the importance of technologies for encouraging breastfeeding can be seen, as they constitute an important tool for establishing a bond, increasing knowledge, as well as an important mechanism for evaluation, whose scenario of encouraging the practice of breastfeeding reaffirms the relevance of nurse. This professional is a great articulator, involving the mother, child, the child’s father or mother’s partner, family members, people close to them^(5,32)^, in the institution of this very important practice, in favor of improving indicators regarding the practice of breastfeeding^(29)^.

However, just the adoption of technologies linked to assistance practice is not enough to encourage breastfeeding, and attention has to be directed to other aspects, which may also be necessary, such as professional qualification for better application of the resource and raising of awareness to recognize the specificities present in each field.

For all these reasons, it is clear that the development and implementation of health technologies are fundamental as a strategy to support care actions carried out by health professionals, and in particular the work carried out by nurses, especially in relation to breastfeeding promotion.

In summary, as a limitation of this study, there is a lack of national research that highlights the use of technologies by nurses to promote breastfeeding, aiming to produce a panoramic view of the use and importance of these technologies in health. Considering several Brazilian health initiatives and policies aimed at promoting, protecting and supporting breastfeeding in Brazil, it was expected to find more national publications on the use of technologies for these purposes.

Furthermore, another limitation is that the articles addressing the topic are unavailable in full. Thus, even though there is a diversity of initiatives and public policies aimed at promoting, protecting, and supporting the practice of breastfeeding in the country, it was expected that more national publications on the use of technologies for these purposes would be found.

## CONCLUSION

The results of this review allowed mapping breastfeeding promotion technologies, as well as categorizing them. Therefore, it was found in this study that technologies related to bonding are the most used by nurses, being a favorable result, since this practice is permeated by the relational dynamics between the healthcare user and the nurse.

It was also observed that all the technologies mentioned in the articles and the combination of different technologies contributed to strengthening the promotion, protection, support, and maintenance of breastfeeding. This review also found that hard technology was the most used as a facilitating strategy to promote breastfeeding, followed by soft technology. However, soft-hard technology was used in few studies and its use by nurses should be encouraged. Finally, it was also observed that no article used assistance technologies, which highlights the need for their implementation in assistance.

Consequently, it is believed that this study, by giving visibility to the technologies used by nurses to promote breastfeeding, can motivate the implementation of more strategies, as well as the innovation of new technologies, with the aim of improving the prevalence and duration of breastfeeding.
